# Dietary assessment tools for children and adolescents in Latin America: a scoping review

**DOI:** 10.1017/jns.2026.10116

**Published:** 2026-06-17

**Authors:** Milca Vidal, Muhammed Iqbal, Janet Cade, Camilla Nykjaer

**Affiliations:** 1 Food Science and Nutrition, University of Leeds Faculty of Environment, UK; 2 Clinical nutrition, Politeknik Negeri Jember, Indonesia; 3 Nutrition Epidemiology Group, University of Leeds, UK; 4 School of Biomedical Sciences, University of Leeds Faculty of Biological Sciences, UK

**Keywords:** Adolescents, Children, Dietary assessment tool, Latin America, Scoping Review

## Abstract

Accurately assessing dietary intake in children and adolescents is essential for understanding dietary patterns and informing public health strategies. In Latin America, rapid nutrition transitions and increasing childhood obesity highlight the need for culturally appropriate, validated dietary assessment tools (DATs). However, methodological challenges and limited regional data hinder effective dietary surveillance. This scoping review identified and characterised DATs used among children and adolescents (5–18 years) in Latin America, examining tool types, features, validation and regional coverage. Following Joanna Briggs Institute and PRISMA-ScR guidelines, comprehensive searches were conducted in EMBASE, Web of Science, PubMed, and LILACS (April 2024) in English, Spanish, and Portuguese. Eligible studies included original research developing, validating, or applying DATs in Latin American populations. Of 13,946 records screened, 105 reports met the inclusion criteria. Brazil and Mexico contributed the most studies, while six countries (Paraguay, El Salvador, Nicaragua, Panama, Honduras, and Belize) had none. Forty-three DATs were identified, 77% of which were food frequency questionnaires (FFQs). Half targeted adolescents, 39% children, and 11% both groups. Most were interviewer-administered (58%) and applied in person (49%), with only 19% conducted online, reflecting regional digital limitations. Validation was reported for 70% of tools, primarily against 24 HR. The DAT landscape in Latin America remains dominated by FFQs and traditional administration methods, with limited use of digital platforms. Developing age-appropriate, validated and culturally adapted digital DATs is essential to strengthen dietary surveillance and guide effective nutrition policies across the region.

## Introduction

Children’s dietary intake is a fundamental determinant of their immediate and long-term health, influencing nutritional status and the risk of developing diet-related non-communicable diseases (NCDs).^(1)^ The assessment of diet in young populations is crucial for understanding dietary patterns, identifying nutritional deficiencies, and developing public health strategies.^(2,3)^ However, accurately measuring dietary intake in children presents unique challenges due to their developing cognitive abilities, reliance on surrogate reporters, and limitations in portion size estimation.^(4,5)^

Many countries in Latin America, like Brazil, Mexico, Chile, Colombia and Argentina, are undergoing a rapid nutrition transition, characterised by a shift from traditional diets based on foods such as beans, corn, rice, fresh vegetables, and fruits to highly processed, energy-dense foods high in sugar, saturated fats, and refined carbohydrates.^(6)^ This transition toward a more Westernised dietary pattern has contributed to rising rates of overweight, obesity, and NCDs such as cardiovascular diseases and type 2 diabetes, especially in young populations.^(7)^ National surveys have documented increases in childhood and adolescent obesity rates in these countries over recent decades. For example, in Mexico, the National Health and Nutrition Survey (ENSANUT) data indicates that the prevalence of obesity among school aged children rose from 34% in 2012 to 37% in 2020.^(8)^ In Brazil, a study using the Food and Nutrition Surveillance System (SISVAN) analysed trends in child and adolescent obesity between 2013 and 2022. The study found that obesity prevalence among children aged 5 to 9 years increased from 12% to 16% (*p* = 0.007), and among adolescents aged 10 to 19 years increased from 6% to 13% (*p* < 0.001).^(9)^ Similarly, in Argentina, analyses of the Global School-based Student Health Surveys conducted in 2007, 2012 and 2018 showed that among adolescents aged 13–15 years, the prevalence of overweight/obesity increased from 22% to 35% in boys and from 12% to 26% in girls. Despite the urgency of monitoring dietary behaviours in this region, the availability of culturally and methodologically appropriate dietary assessment tools (DATs) for children and adolescents remains limited. A lack of validated instruments compromises the ability to draw reliable conclusions about diet-disease relationships, ultimately affecting the effectiveness of public health interventions.

Several dietary assessment methods exist, including food frequency questionnaires (FFQs), 24-h dietary recalls (24 HR), and food diaries, each with distinct strengths and limitations. FFQs are widely used in large-scale studies due to their ability to capture habitual intake over extended periods; however, they must be tailored to specific study populations and research aims, as dietary patterns can vary with age, ethnicity, culture, personal preferences, and socioeconomic status.^(10,11)^ They also tend to misestimate intake of nutrients, particularly those contributed in mixed dishes, compared to other tools.^(12)^ In contrast, 24 HR provide detailed short-term dietary data but require multiple recalls to reliably estimate usual intake for the individual.^(13)^ Technological innovations, such as mobile applications, digital photography, and automated food recognition systems, have been developed to facilitate reporting and enhance data accuracy.^(14–16)^ However, all tools still face methodological challenges, including measurement errors, dependence on self-reporting, the suitability of culturally appropriate food composition tables for nutrient estimations, and the need for validation in diverse populations.^(17,15)^ Previous reviews of DATs in children and adolescents have highlighted the strengths and limitations of existing methodologies.^(5,18)^ Some reviews have focused exclusively on specific population groups of children or adults,^(19,20)^ particular dietary assessment methods,^(21)^ or validation studies,^(22)^ while other reviews have lacked standardised methodologies or regional specificity.^(23)^ Furthermore, limited research has examined DATs specifically in the Latin American context, where dietary habits, food availability, and cultural influences differ from other regions. Addressing these gaps requires a comprehensive evaluation of the tools currently used to assess dietary intake in children in Latin America.

A scoping review was deemed the most suitable approach to address these gaps, as it allows for a broad exploration of the existing DATs, their characteristics, validity, and applicability within the region. Unlike systematic reviews, which focus on specific research questions and study designs, scoping reviews provide an overview of the available evidence, identify knowledge gaps, and offer insights into future research directions.^(24)^ Therefore, this review aimed to map the current landscape of DATs for children in Latin America, evaluating their characteristics, strengths and limitations, to improve the quality of dietary information and nutritional epidemiology research.

## Methods

### Study design

This scoping review was conducted following the methodology outlined in the Joanna Briggs Institute Reviewer Manual.^(25)^ The reporting of the review adheres to the PRISMA-ScR (Preferred Reporting Items for Systematic Reviews and Meta-Analyses Extension for Scoping Reviews) checklist to ensure transparency and completeness in the review process^(26)^ (Appendix 1). As recommended for scoping reviews, formal critical appraisal was not undertaken because the purpose was to map the evidence base rather than to estimate effect or make causal inferences.^(27,28)^ The study protocol was registered with the Open Science Framework (https://doi.org/10.17605/OSF.IO/9S8YG) before the initiation of data collection in March 2024 and updated in November 2024.

To guide the search strategy, the PCC (Population, Concept, and Context) framework was employed and identified as:

- Population: Children and adolescents aged 5 to 18 years.
- Concept: DATs, encompassing both traditional and technology-based methods.
- Context: The Latin American region, considering studies conducted in any country within this geographical area (Mexico, Costa Rica, El Salvador, Brazil, Guatemala, Nicaragua, Panama, Colombia, Argentina, Peru, Chile, Paraguay, Uruguay, Ecuador, Bolivia, Venezuela, Honduras, Belize).

### Search strategy

A systematic literature search was conducted in four databases to ensure a comprehensive identification of relevant studies. The selected databases included EMBASE, Web of Science, PubMed (including MEDLINE), and LILACS (Latin American and Caribbean Literature in Health Sciences). The search was conducted using the following interfaces: Ovid, Clarivate Analytics, and the National Library of Medicine. The final search strategy (appendix 2) incorporated controlled vocabulary and free-text terms to capture a broad range of studies related to DATs for Latin America populations.

The search terms were carefully constructed to include key concepts related to DATs, food intake evaluation, and nutrition assessment tools, with specific filters to ensure relevance to Latin American populations. Age-specific terms were not included, as the search strategy was developed to support a broader evidence review that encompasses both adult and youth populations. For the current scoping review, however, only studies involving children and adolescents were included based on eligibility criteria applied during screening. The search strategy was designed to cover studies that assessed dietary intake using both traditional and technology-based tools.

To complement the database search, additional sources were identified through cross-referencing by manually screening the reference lists of relevant studies. In cases where additional data or supplementary materials were required for inclusion, the corresponding authors were contacted. If methodological details or study results were unclear, further clarification was sought from the authors. Contact attempts were made after the initial screening, with a two-week response window before considering the attempt unsuccessful.

The search was limited to studies published in English, Spanish, and Portuguese, and no restrictions were applied regarding the publication date. A detailed breakdown of the query strings used in each database is provided in appendix 2.

### Eligibility criteria

The inclusion and exclusion criteria for this scoping review were established *a priori* to ensure a systematic selection of relevant studies.

#### Inclusion criteria

This review considered original studies that assessed dietary intake using traditional or technology-based DATs in children and adolescents in Latin America, with the target population defined as 5–18 years. Studies involving broader or mixed-age populations were eligible if they reported separate results for the target age group. In cases where data could not be disaggregated, studies with slight age overlap at the margins (e.g., ages 4 or 19) were included, provided the study population remained substantially aligned with the review’s focus on childhood and adolescence. Studies using, developing, validating, or comparing DATs, including manual versus digital versions, were eligible.

Additionally, studies with broader objectives, such as examining the association between dietary intake and health outcomes (e.g., obesity, malnutrition, dietary patterns) were included if they described the DAT used. Eligible studies had to be published in English, Spanish, or Portuguese and available up to November 2024.

For the purposes of this review, a distinct DAT was defined as a unique dietary tool with a clearly identifiable structure and assessment design. Repeated use of the same instrument across different studies was counted as a single DAT. Adapted or revised instruments were classified as distinct only when studies reported substantive modifications affecting the content or structure of the tool, such as changes in the food item list, portion size estimation method, reference period, administration procedure, or functionality beyond mode of delivery alone. Differences limited to format only (e.g., paper versus digital) were not considered sufficient to define a new DAT unless accompanied by substantive methodological changes. This classification was applied consistently during data extraction and synthesis.

#### Exclusion criteria

Studies were excluded if they focused on non-Latin American populations or on Latin Americans living outside the region. Articles were also excluded if they assessed dietary intake using tools designed exclusively for clinical populations (such as patients with diabetes, cancer, or other medical conditions) or for athletes. Similarly, studies that employed DATs limited to a single food or nutrient (e.g., calcium, sugar, or sodium) were not considered. Research that used DATs solely for secondary purposes, such as dietary education, food preference assessment, or promotion of healthy habits, was also excluded. In addition, study protocols, conference abstracts, and review articles were not eligible, and studies that did not provide sufficient details regarding the dietary assessment tool for data extraction such as the method or period of assessment, description of the tool, reference or source of the tool (e.g. original publication or validation study), were excluded.

Screening and data extraction

#### Initial selection: Title and abstract screening

All records identified through the systematic search were first exported into an EndNote library, where duplicate records were removed. The library was then exported into SR-Accelerator, a tool used to facilitate systematic reviews.

Two researchers (M.I. & M.V) conducted the title and abstract screening independently, applying the pre-established inclusion and exclusion criteria. After individually selecting their final list of reports, the researchers compared their selections within SR-Accelerator to identify matches. Any discrepancies between selections were discussed until a consensus was reached regarding the inclusion or exclusion of specific studies.

#### Final selection: Full-text screening

For the records that passed the title and abstract screening, full-text reports were retrieved using EndNote’s full-text search functionality, or when not available through EndNote, full-text files were searched in PubMed and Google Scholar. During the full-text screening, the inclusion and exclusion criteria were reapplied to ensure that all retained studies met the predefined eligibility.

#### Data extraction

The final dataset of included studies underwent data extraction by the primary researcher (M.V.), focusing on key details regarding the DATs. The extracted data included:

- Study characteristics: reference, study design, sample size, country, region, sex, age group.
- Characteristics of DATs (when applicable): name of the tool, type of technology, dietary assessment method used, administration method, person reporting intake, period of assessment, validation method, language, age group targeted, setting where the tool was applied, food composition database (FCDB) used, number of items in the tool, method for portion estimation.
- Results and outcomes: food groups assessed, energy, macro and micronutrient intake recorded.
- Validation outcomes of relative validity (when applicable): kappa value (agreement), Pearson correlation coefficient (r) (energy-adjusted and deattenuated), intraclass correlation coefficient, exact agreement (%) (correct classification of quartiles), and gross misclassification (% of subjects misclassified).

### Data analysis and synthesis

A narrative synthesis of the selected studies was used to map the existing literature. Absolute and relative frequencies of key characteristics of the DATs, including their methods, validation processes, and population coverage were calculated. Data analysis was performed using Microsoft Excel and R (version 4.3.3) to generate descriptive statistics, such as proportions and distributions and for data visualisation. As this was a scoping review intended to map the range and characteristics of available DATs, formal methodological quality appraisal was not undertaken. Nonetheless, when summarising validation outcomes, attention was given to differences in reporting completeness, validation design, reference methods, and transparency of outcome reporting, and findings were interpreted with appropriate caution.

## Results

A total of 13,946 records were retrieved from the databases, yielding 11,488 records after excluding duplicates. The search strategy was intentionally broad, as it was developed as part of a protocol for a larger evidence review that included both adult and youth populations. As a result, many of the retrieved records involved studies with adult participants. For this scoping review, only studies involving children and adolescents were eligible, and studies including adult populations were excluded during the screening process.

**Figure 1.**
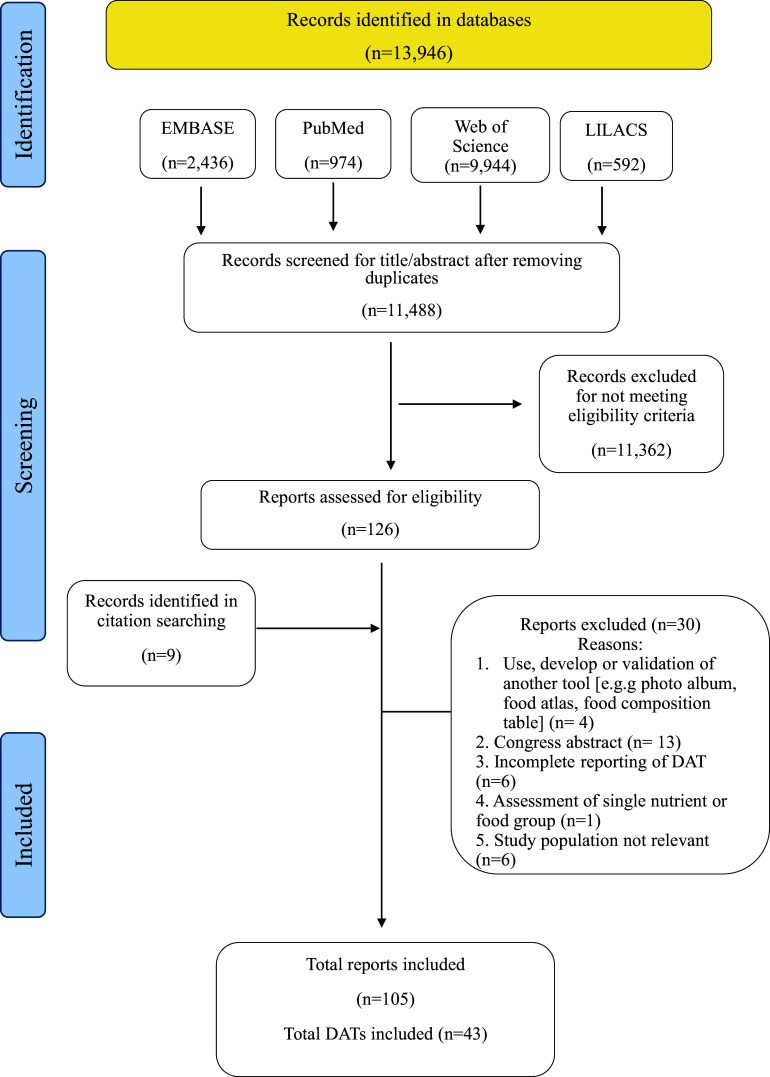
Figure 1 long description. PRISMA-ScR Flow Diagram describing the scoping review process.

Titles and abstracts were screened based on the inclusion and exclusion criteria, resulting in 126 reports eligible for full-text review. After full-text assessment, 30 reports were excluded for reasons such as using, developing, or validating other tools than a DAT (*n* = 4), being conference abstracts (*n* = 13), incomplete reporting of the DAT (*n* = 6), assessment of single nutrients or food groups (*n* = 1), or focusing on a different or clinical population (*n* = 6). Additionally, nine new reports were identified through cross-referencing, leading to a final inclusion of 105 reports in the review representing 43 different DATs (Figure 1).

The results are presented in four parts: the overall characteristics of included reports, the distribution and characteristics of identified DATs, patterns in validation outcomes, and descriptive variation in reported energy intake. Throughout, emphasis is placed on findings with relevance to methodological gaps and future tool development in the region.

### Main characteristics of included reports

The included literature was geographically concentrated and methodologically dominated by cross-sectional studies, with Brazil and Mexico accounting for most of the available evidence. Of the 105 reports included in this review 66 (63%) use cross-sectional designs. Twenty-eight (27%) were validation studies and seven (7%) were national survey reports. Of these seven, four described results from nationwide surveys in Brazil, while the other three were based on the national dietary survey of Mexico. The remaining reports consisted of longitudinal, development, adaptation, and reproducibility studies (*n* = 4, 4%).

Most of the studies were conducted in Brazil (*n* = 67, 64%) and Mexico (*n* = 12, 11%), followed by Argentina, Guatemala, Colombia, Chile, Peru, Venezuela, Uruguay, Ecuador, Bolivia, and Costa Rica. No studies were identified from El Salvador, Nicaragua, Panama, Paraguay, Honduras, or Belize. Regarding the settings of the studies, 31(30%) were conducted in urban areas, 7(7%) in rural areas, and 17(16%) included both urban and rural populations while 50(48%) did not specify the setting of the study. The age of participants ranged from 5 to 18 years in 74(70%) reports. Six reports (6%) included participants aged 4 years, and 24 reports (23%) included participants up to 19 years. Energy and nutrient intake were reported in 57 publications (54%), while the rest reported dietary patterns (*n* = 7, 7%), food group intake (*n* = 9, 10%), adequacy or quality of diet (*n* = 4, 4%) and validation outcomes (*n* = 28, 27%). Two of the validation studies included in this review focused specifically on the development and validation of the FFQ used in the Mexican national survey. The majority of studies took place in schools, homes, and health centres (Supplementary material table 1).

**Figure 2.**
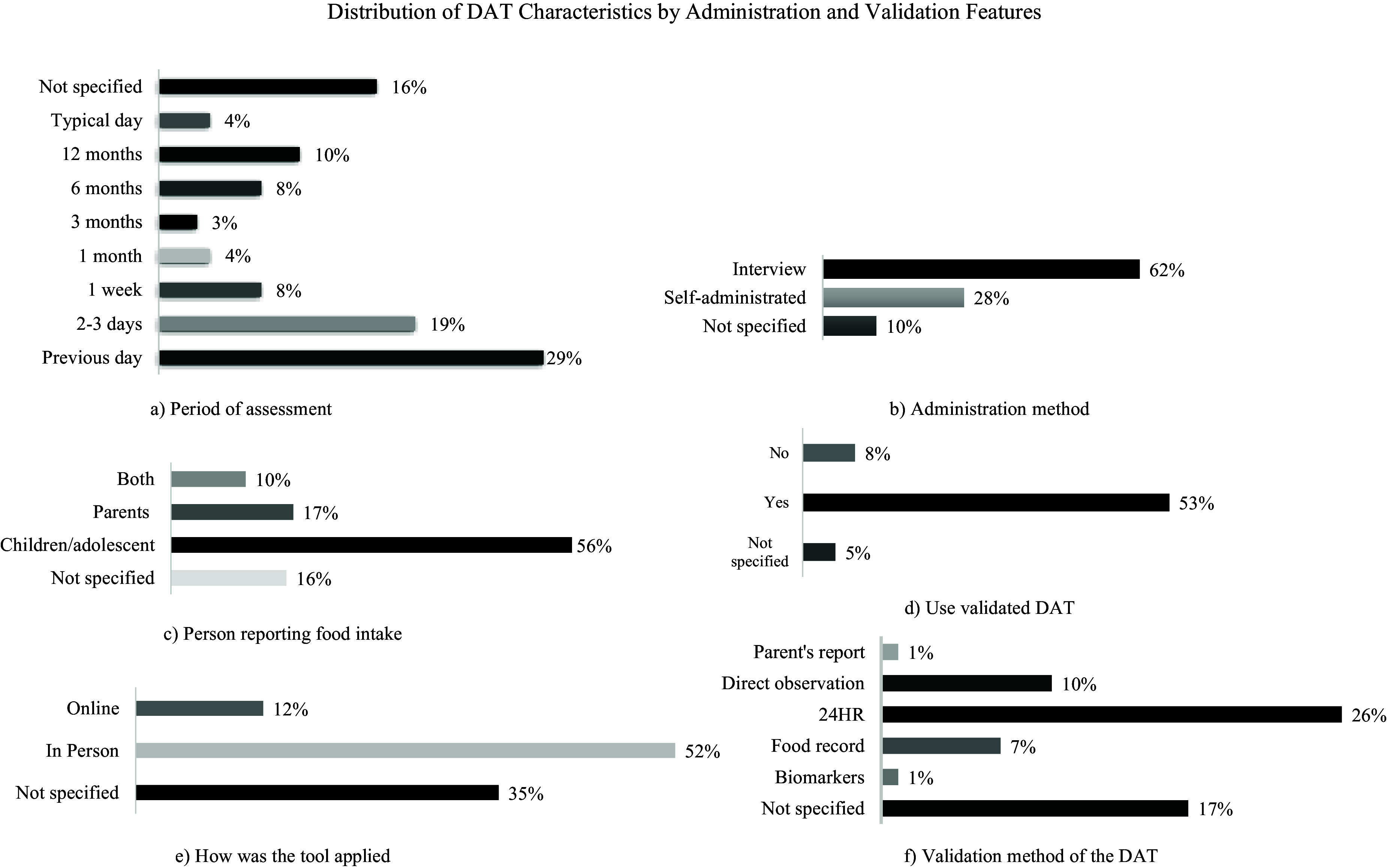
Figure 2 long description. Detailed summary of DATs characteristics used to evaluate food intake among children and adolescents in the included reports (*n* = 105).

Fifty-one reports (49%) used FFQs as a DAT, followed by 24 HR in 32 (30%). Other methods used were food records (*n* = 9, 9%), illustrated food checklist (*n* = 12, 11%) and food questionnaire (*n* = 1, 1%). Data collection methods varied, with interviewer administration being the most prevalent, reported in 65(62%) reports, while self-administered tools were employed in 29(28%), particularly for FFQs and food records (Figure 2b). The most common person reporting the intake was the child or adolescent themselves (*n* = 59, 56%), especially in self-administered methods. In 18(17%) reports the parents, tutors, or persons responsible for the child’s diet provided the information. In 11(10%) reports, both the child and a parent or tutor were asked about the food consumption (Figure 2c). In 55 (52%) reports, dietary data were collected in person, 13(12%) online and only two reported using phone calls to collect dietary data on a second occasion (Figure 2e).

### Characteristics of the DATs

A total of 43 distinct DATs were identified across the included reports, with distinctness defined at the level of unique instrument identity rather than number of applications across studies. Repeated use of the same instrument was counted once. The characteristics of the DATs are presented in Table 1. Of these, 33(77%) were FFQs, four (9%) were illustrated food checklists or questionnaires, four (9%) were based on the 24 HR method, one DAT (2%) was the food record, and one (2%) was a general food questionnaire. The latter consisted of 12 questions that qualitatively assessed food consumption across multiple food groups without a defined reference timeframe. In terms of validity, 30(70%) had been validated, five (11%) did not report validation status of the DAT, three (7%) were not validated and five tools (11%) were validated or developed in a different population (adults and other countries) but not in children or adolescents. The 24 HR and food record were identified among the DATs, counted as one of each, however, they were used as tools to assess diet and did not report validation. Of the remaining tools, the validation methods of the DATs included comparison against a 24 HR (*n* = 16), food records (*n* = 5), direct observation (*n* = 3), comparison with parent’s report (*n* = 1) and biomarkers (*n* = 1). The latter included creatine, myristic acid (C14:0), alpha linolenic acid (ALA), eicosapentaenoic acid (EPA), b-carotene, retinol, a-tocopherol, 25-OH-vitamin D, thiamine, riboflavin, nicotinamide, pantothenic acid, pyridoxal 5’-phophate, folate and vitamin B12.

**Table 1.** Summary characteristics of the 43 DATs identified Table 1 long description.

Method used	Country	Sample size	Validity (validation method)	Administration method	Person reporting	Period of assessment
24 HR^(29–32,34,54–79)^ n = 4 tools	BRA, MEX, COL, CHL, VEN, ARG, PER, URY, BOL	41 to 71,971	NS	Interview in person (*n* = 1), online (*n* = 1) Self-administered in person (n = 0), online (n = 2)	Parents, (*n* = 1) Child or adolescent, (*n* = 2) Both, (*n* = 1)	1 to 3 days
FFQ^(33,36,80–125)^ n = 33 tools	BRA, MEX, COL, ECU, ARG, PER, BOL, GTM, CHL, URY	46 to 8,716	Validated by 24 HR (*n* = 16), food record (*n* = 5), biomarkers (*n* = 1), direct observation (*n* = 1), NS (*n* = 4) Not validated, (*n* = 3), NS (*n* = 2)	Interview: in person (*n* = 13), online (*n* = 1), NS (*n* = 13) Self-administered in person (*n* = 3), online (*n* = 1), NS (*n* = 2) NS, (*n* = 3)	Parents, (*n* = 7) Child or adolescent, (*n* = 16) Both, (*n* = 5) NS, (*n* = 5)	1 week to 12 months
Food record^(72,126–133)^ n = 1 tool	BRA, MEX, CRI, GTM	90 to 5975	NA	Self- administered in person	Child or adolescent	1 to 3 days
Illustrated food checklist ^(134–146)^ n = 4 tools	BRA, ARG	32 to 6797	Validated by direct observation (*n* = 3), parent’s report (*n* = 1)	Self-administered in person (n = 2), online (*n* = 3)	Child or adolescent (*n* = 4)	Previous day
Food questionnaire *n* = 1 DAT^(147)^	CHL	90	NS	Interviewed in person	Children or adolescent	Typical day

BRA = Brazil, MEX = Mexico, CHL = Chile, COL = Colombia, PER = Peru, ARG = Argentina, ECU = Ecuador, CRI = Costa Rica, GTM = Guatemala, VEN = Venezuelan, URY = Uruguay, NS = not specified, NA = not applicable.

*n* = number of reports using the DAT.

Of the 43 DATs identified in the 105 articles, two represented substantively modified versions of an existing tool, involving both a change in delivery format and changes in portion estimation, and were therefore classified as distinct instruments. Twenty-seven percent of the DATs were self-administered, 58% used an interviewer for its application, 15% did not specify how the tool was applied. Forty-nine percent were applied in person while 19% were online. Of the remaining DATs, 32% did not specified the administration method.

**Figure 3.**
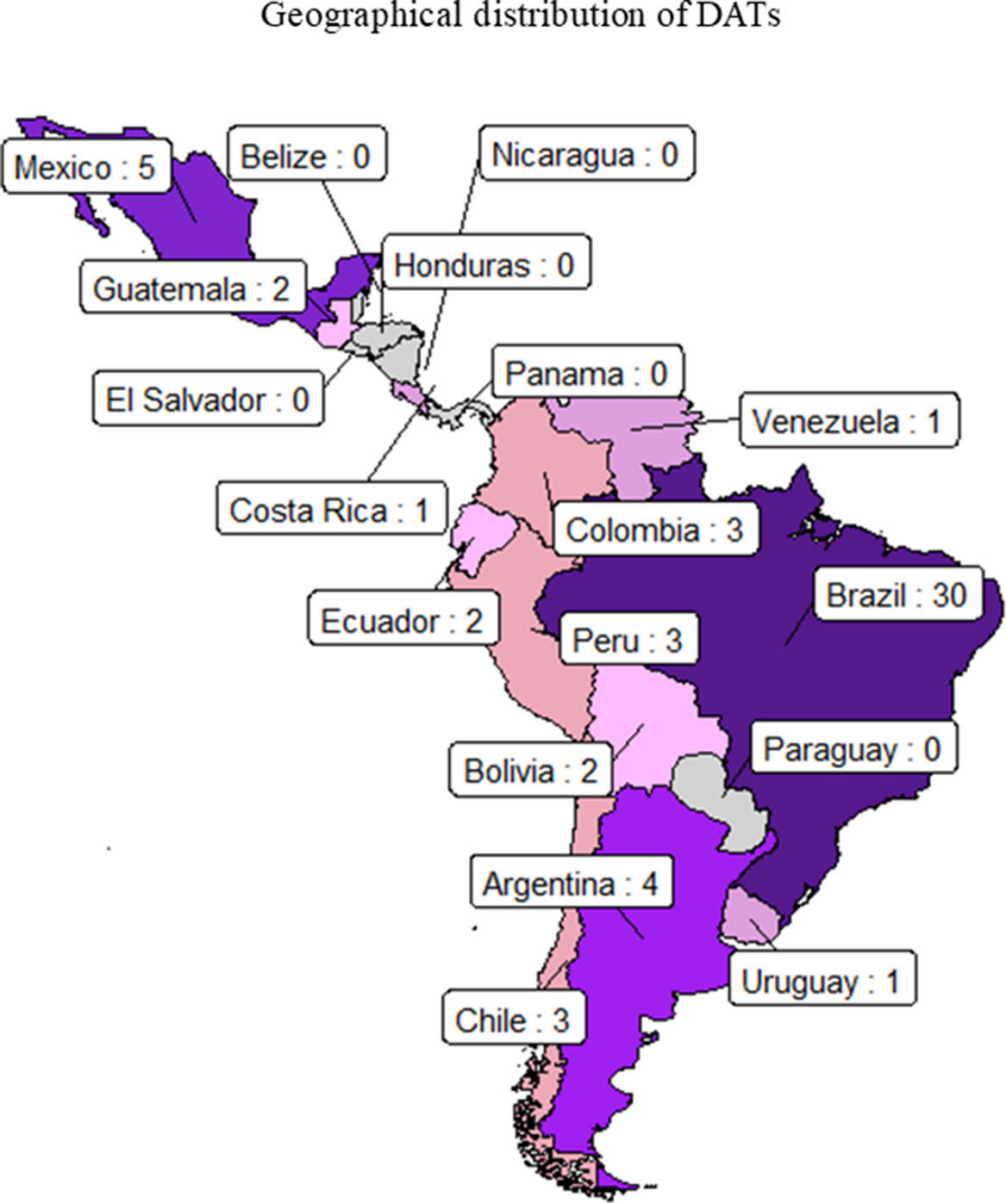
Figure 3 long description. Number of dietary assessment tools in Latin America distributed geographically per country.

The geographical distribution of DATs identified is shown in Figure 3. Some DATs were applied or validated in more than one country (e.g. 24 H and food record), therefore, the distribution represents the total number of DATs available for use within each country rather than the unique tools. The Latin America map shows that Brazil has the highest number of DATs (*n* = 30), followed by Mexico (*n* = 5), Argentina (*n* = 4), Peru (*n* = 3), Colombia (*n* = 3), Chile (*n* = 3), Bolivia (*n* = 2), Guatemala (*n* = 2) and Ecuador (*n* = 2). Only one DAT was identified in Venezuela, Costa Rica, and Uruguay whereas none were found in Paraguay, El Salvador, Nicaragua, Panama, Honduras or Belize.

Regarding the quantification of dietary intake, semi-quantitative methods were the most widely used, applied in 50% of the DATs, while qualitative approaches were used in 30% and quantitative methods in 20%. The qualitative tools did not provide portion size or frequency information; they only recorded whether specific food items were consumed, using illustrated checklists (circling images) or yes/no food questionnaires. The methods for portion size estimation in the semi-quantitative DATs varied, with 40% of the studies relying on standard portions derived from 24h recalls or standard household measures, 30% using images such as atlases, photo albums, or visual guides, and the remaining 30% did not specify the method used.

**Figure 4.**
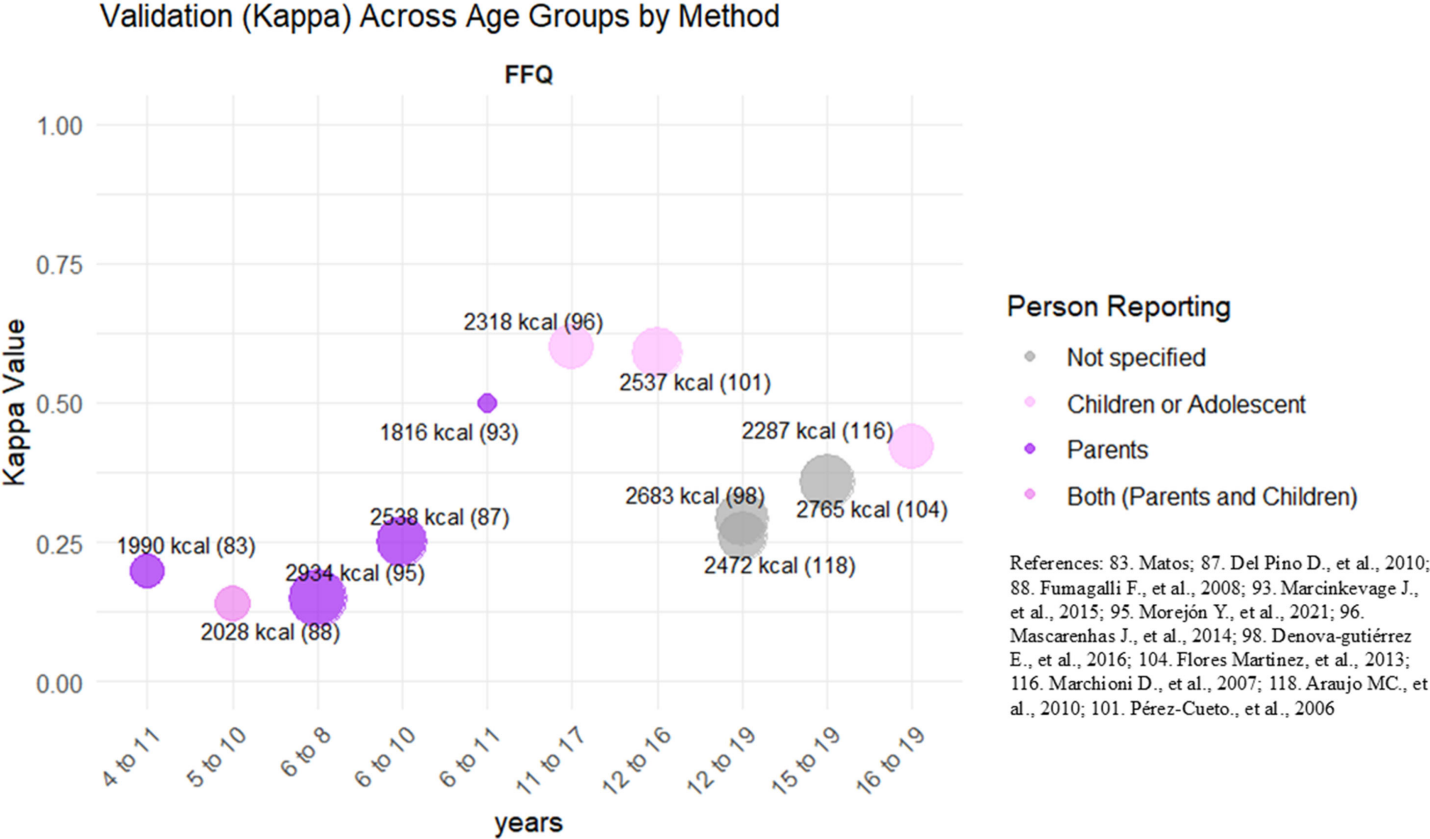
Figure 4 long description. Validated DATs: mean Kappa coefficients by age group (Bubble size = Mean energy intake). Colour intensity represents the person reporting the food intake. Validation findings are based predominantly on relative validity studies using heterogeneous reference methods.

Among the validated DATs, FFQs were the most used method and were predominantly completed by children or adolescents (*n* = 18, 60%), followed by reporting from parents (*n* = 7, 23%) or both parent and child or adolescent (*n* = 5, 17%); the illustrated food checklist and questionnaire were also primarily completed by children or adolescents. Figure 4 summarises validation findings across age groups based on reported kappa values and mean energy intake. All DATs included in this comparison were FFQs. In studies involving older age groups (11–17 years), reported kappa values tended to be higher than in studies targeting younger children (<10 years), in which values were often below 0.5. A similar pattern was observed in some FFQs completed by children or adolescents, which showed relatively higher reported kappa values than those completed by parents. Mixed reporting by parent and child/adolescent also showed moderate agreement in some studies involving older age groups. Overall, reported kappa values across FFQs ranged from 0.1 to 0.6, reflecting low to moderate agreement with the reference methods used in the included studies.

**Figure 5.**
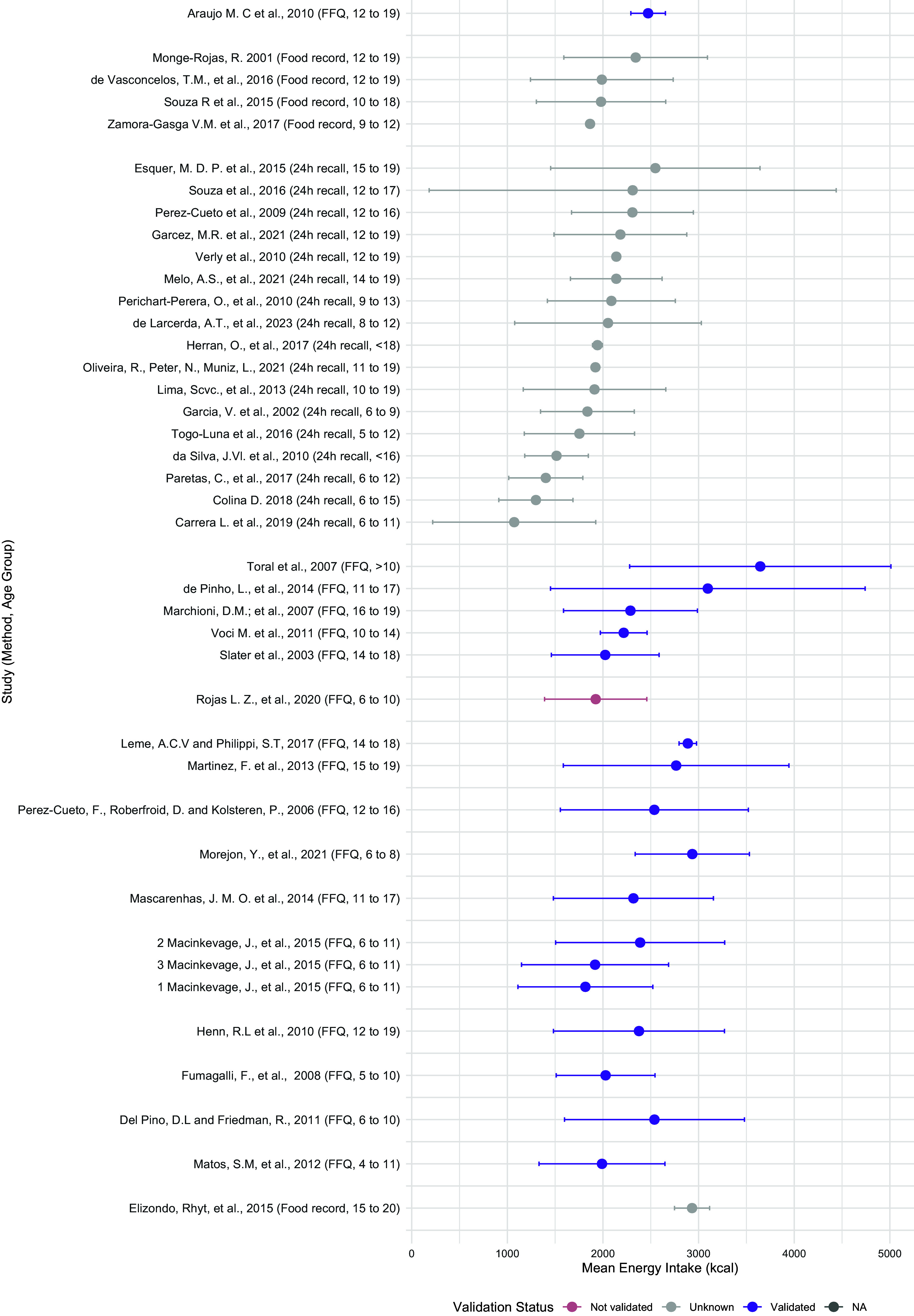
Figure 5 long description. Forest plot of mean energy intake reported in studies using different DATs (type of DAT and age range in brackets) and validation status.

The variability of energy intake reported as mean daily energy intake ± standard deviation (SD) across DATs is illustrated in Figure 5. Studies employing 24 HR (*n* = 17) reported mean energy intakes ranging from 1072 kcal^(29)^ to 2548 kcal^(30)^ with an overall mean of 1947 kcal. Standard deviations varied considerably (36 to 2128 kcal) reflecting differences in population characteristics, recall quality, possibly underreporting and study settings (e.g. sample size). Lower values were more frequently observed in studies involving younger children aged 6–11 years using 24 HRs.^(29,31–34)^ Studies using FFQs (*n* = 19) showed mean energy intake values ranging from 1816 kcal^(35,36)^ to 3645 kcal,^(36)^ with a weighted average of 2372 kcal, whereas food record studies (*n* = 5) ranged from 1865 kcal to 2932 kcal, with a mean intake of 2221 kcal and generally large SDs (>700 kcal).

## Discussion

This scoping review aimed to identify and characterise the tools to assess the diet in children and adolescents across Latin America. Specifically, it sought to map the types, features, and methodological rigour of DATs applied in these populations and context. Overall, the review highlights three major priorities for the region: broader geographic coverage beyond Brazil and Mexico, stronger and more transparent validation practices, and greater development of context-appropriate digital tools for children and adolescents. These priorities are directly relevant for improving the quality, comparability, and policy usefulness of dietary data in Latin America.

From 105 reports, a total of 43 distinct DATs were identified and analysed. While the geographic scope of the review was Latin America, most tools originated from Brazil and Mexico, making these countries the central focus of the findings. This regional concentration underscores the variability in dietary surveillance capacity across Latin America, with limited representation from Central America children and adolescents. For instance, countries such as El Salvador, Nicaragua, Venezuela, Honduras, and Panama have documented facing nutritional challenges where malnutrition coexist with raising rates of overweight and obesity. In 2019, the National Health Survey of Panama (ENSPA) reported a combined prevalence of overweight and obesity of 37% in children aged 5–9 years, while among adolescents aged 10–14 years 22% were overweight and 15% had obesity; concurrently, the prevalence of undernutrition in children under 5 years was 15% (95% CI: 13.4, 17.3).^(37,38)^ Similarly, El Salvador experienced a marked burden of excess weight among school-age children in the 2015–2016 National School Height and Weight Census.^(39)^ Using the WHO-2007 growth reference, 30% of children aged 6-9 years were classified as overweight or obese.^(39)^ In the younger group, UNICEF Progress Assessment, 2018–2022 reports a prevalence of stunting and excess weight of 15% and 7% among children <5 years, respectively.^(40)^ These reports highlight the double burden of malnutrition in the region, however, despite existing anthropometric monitoring, limited dietary intake data is available for a comprehensive understanding of these nutrition outcomes.

## Types and characteristics of DATs

FFQs dominated the landscape, accounting for 77% of all identified tools. The high prevalence of FFQs is consistent with their logistical advantages in large-scale, low-resource settings, such as lower respondent burden, cost-effectiveness, and ease of administration.^(10)^ While food records and 24 HR recalls may offer more accurate, real-time intake data, their high variability and participant burden make them less commonly used in large-scale studies involving adolescents.^(41,42)^ These findings emphasise the methodological impact on energy intake reporting in young populations. Also, it is important to note that FFQs have historically been the primary method in large cohort studies globally, due to these same strengths.^(12,43)^ However, the continued reliance on FFQs in Latin America contrasts with recent trends in high-income countries, where 24 HR recalls and digital self-report tools are becoming more widely adopted to improve precision and respondent engagement.^(16,44)^

In contrast to FFQs, only 9% of the DATs used 24 HR and another 9% employed qualitative tools such as illustrated checklists. While qualitative tools may reduce cognitive demands for younger children, their inability to quantify intake restricts their utility for nutritional epidemiology and public health planning. Semi-quantitative methods to estimate the amount of food consumed by children or adolescents were common (50%), with 40% using standard portion size estimations (kitchen measurements), 30% using food images, and 30% failing to specify the estimation approach.

## DAT validation practices

Validation practices varied widely across studies. Of the 43 DATs, 70% reported a validation status, most often as relative validity in comparison against 24 HR recalls. Only one tool used biomarkers as a reference method, reflecting a gap in the use of objective validation approaches. However, it is important to recognise that biomarkers have limitations, they can be influenced by factors beyond dietary intake, including genetics, metabolic variations, and health status. Moreover, biomarker-based validation is often costly, logistically complex, and less feasible in large-scale or low-resource settings.^(45)^ Four DATs did not specify the validation method at all, indicating a lack of reporting rigour that could compromise interpretability.

Across age groups, parent-reported FFQs were more common in studies involving children under 11 years, while self-report or combined parent–child report tools were used more frequently for older adolescents. Interestingly, relatively higher agreement levels (e.g., kappa values) were observed in validation studies when children or adolescents reported the intake. This could indicate that children and adolescents may have better knowledge of what they eat and are able to provide more details about their food consumption.^(46,47)^ Although some studies reported relatively higher kappa values for FFQs completed by children or adolescents than for those completed by parents, this pattern should be interpreted with caution.^(47,48)^ The validation evidence identified in this review was based predominantly on relative validity designs, most commonly comparisons with other self-reported dietary methods rather than objective gold-standard measures. In addition, reported kappa values were generally low to moderate, and differences across studies may reflect variation in age group, administration procedures, reference methods, recall demands, and context of food consumption rather than true differences in reporting accuracy. Therefore, the findings suggest an observed pattern in the available literature, but they do not establish that child or adolescent self-report is inherently superior to parent report. For younger children under 10 years, observer-based or a combined parent–child reporting may offer more reliable intake data, supporting existing evidence that memory and cognitive maturity also impact reporting accuracy.^(2)^

## Digital tools and access barriers

Interviewer-administered tools were most prevalent (58%), followed by self-administered tools (27%), while 15% of studies did not report the administration method. Despite the growing global use of web-based 24 HRs with automated coding, image capture, and mobile apps, only 19% (*n* = 8) of the DATs in this review were administered online. Examples of global research tools in this domain include ASA24, myfood24, and Intake24, which combine automation with user-friendly interfaces to reduce burden and enhance accuracy.^(49,50)^

In Latin America, however, limited online use likely reflects broader infrastructural and socioeconomic challenges. Although smartphone ownership and internet access have grown rapidly across the region, disparities persist by country, income level, and urban–rural location. For instance, while smartphone penetration is above 70% in Brazil and Mexico, it is lower in parts of Central America and among rural populations.^(51,52)^ Moreover, digital dietary tools impose a considerable burden on researchers, requiring access to cloud infrastructure, software customisation, technical support, and trained staff to manage and interpret complex data. These barriers contribute to the slow adoption of digital methods in the region, potentially exacerbating health data inequalities and limiting the scalability of innovative surveillance systems.^(53)^

## Variability in energy intake reporting

The forest plot of mean energy intake from 41 studies revealed substantial heterogeneity, with estimates ranging from approximately 1,200 to over 3,000 kcal/day. This variation reflects multiple contributing factors: differences in DAT design, administration method, age group, and geographic setting, as well as inconsistencies in portion size estimation and energy conversion protocols. Studies using self-report FFQs with older adolescents tended to show the highest intakes (>2,800 kcal), while those employing interviewer-administered 24 HR recalls with younger children reported the lowest (<2,000 kcal). Narrower confidence intervals in some cases suggest greater precision, often associated with larger sample sizes and standardised tools. The values summarised in this review were derived from studies conducted in diverse populations with different age distributions, countries, settings, study objectives, respondent types, and analytic approaches. As such, cross-study differences in mean energy intake cannot be attributed to the DAT alone. Higher or lower reported energy intakes may reflect population characteristics, contextual factors, or study design features as much as, or more than, the method itself. Therefore, the energy intake patterns presented in this review should be understood as descriptive of the existing literature rather than as evidence of the comparative performance of one dietary assessment method over another.

## Strengths and limitations of the review

A key strength of this scoping review lies in its comprehensive approach to mapping DATs used among children and adolescents in Latin America, offering a valuable regional snapshot of methodological practices and gaps. It includes diverse study designs, settings, and age groups, and provides a structured overview of tool features, validation approaches, and digital adoption. Furthermore, its search strategy was comprehensive, addressing the Latin American context by including studies in English, Spanish, and Portuguese. The use of a registered protocol enhanced transparency and methodological rigour, and grey literature was considered through cross-referencing to reduce potential publication bias. To our knowledge, this is the first scoping review to systematically map DATs for children and adolescents across Latin America, highlighting the novelty and relevance of this synthesis.

However, limitations should also be acknowledged. First, the concentration of studies from Brazil and Mexico limits the generalisability of findings to underrepresented countries in Central and South America. Second, not all studies provided sufficient methodological detail, particularly concerning portion size estimation and validation procedures, which may affect interpretability. In addition, no formal quality appraisal was conducted, in line with scoping review methodology. Nevertheless, validation findings were interpreted cautiously by considering the completeness of reporting, the type of reference method used, and the extent of methodological detail provided in the original studies. This was particularly important given the substantial variability in reporting of validation procedures and outcomes across the included literature. Third, information on the underlying food composition tables used for dietary assessment was rarely reported; in many cases these may have relied on USDA or other non-local databases, which could affect accuracy in reflecting regional diets. Finally, variability in how dietary intake and validation outcomes were reported limited the ability to conduct direct comparisons across tools.

## Conclusion

This review highlights the heavy reliance on FFQs, the predominance of interviewer-administered, paper-based tools, and the limited adoption of validated, digital dietary assessment methods among children and adolescents in Latin America. To improve dietary surveillance and inform nutrition policy, future research should prioritise the development of regionally adapted, age-appropriate digital tools, including mobile platforms with culturally relevant interfaces. This includes integrating harmonised FCDBs, enhancing researcher training, and investing in digital infrastructure, to support equitable access to technology, to close data and health equity gaps.

Moreover, expanding the geographic scope of research beyond Brazil and Mexico is essential to ensure regional representativeness and address disparities in dietary data availability. Collaborative initiatives involving governments, academic institutions, and technology developers could accelerate innovation and capacity-building in this space.

Expanding access to and capacity for innovative dietary assessment will be critical for enhancing public health nutrition strategies and achieving better health outcomes for children and adolescents across Latin America.

## Supporting information

10.1017/jns.2026.10116.sm001 Vidal et al. supplementary material Vidal et al. supplementary material

## Supplementary material

The supplementary material for this article can be found at https://doi.org/10.1017/jns.2026.10116.

## Figure 1. Long description

The flowchart begins with the identification stage, where records are identified in databases such as EMBASE, PubMed, Web of Science, and LILACS, totaling 13,946 records. In the screening stage, records are screened for title and abstract after removing duplicates, resulting in 11,488 records. Records not meeting eligibility criteria are excluded, leaving 126 reports assessed for eligibility. Additional records are identified through citation searching, adding 9 more records. In the inclusion stage, 30 reports are excluded for reasons such as using another tool, being a congress abstract, incomplete reporting of dietary assessment tools, assessing single nutrients or food groups, or having a non-relevant study population. The total reports included are 105, with 43 dietary assessment tools included.

Navigate back to Figure 1..

## Figure 2. Long description

The bar graph compares various characteristics of dietary assessment tools (DAT) used to evaluate food intake among children and adolescents. The graph features horizontal bars and is grouped into six sections: period of assessment, administration method, person reporting food intake, use of validated DAT, how the tool was applied, and validation method of the DAT. The x-axis represents the percentage distribution, while the y-axis lists the categories. Key labels include ‘Not specified’, ‘Typical day’, ‘12 months’, ‘6 months’, ‘3 months’, ‘1 month’, ‘1 week’, ‘2-3 days’, ‘Previous day’ for the period of assessment; ‘Interview’, ‘Self-administered’, ‘Not specified’ for the administration method; ‘Both’, ‘Parents’, ‘Children/adolescent’, ‘Not specified’ for the person reporting food intake; ‘No’, ‘Yes’, ‘Not specified’ for the use of validated DAT; ‘Online’, ‘In Person’, ‘Not specified’ for how the tool was applied; and ‘Parent’s report’, ‘Direct observation’, ‘24HR’, ‘Food record’, ‘Biomarkers’, ‘Not specified’ for the validation method of the DAT. The color scheme includes black, gray, and light gray bars, each representing different data points. Notable trends include a high percentage of interviews (62%) and children/adolescents reporting their own food intake (56%). The graph represents data from 105 included reports. All values are approximated.

Navigate back to Figure 2..

## Table 1. Long description

The table presents a summary of the characteristics of 43 distinct dietary assessment tools (DATs) identified across various studies. It includes details such as the method used, countries where the tools were applied, sample sizes, validity methods, administration methods, person reporting, and period of assessment. The methods used include 24-hour recall, food frequency questionnaires, food records, illustrated food checklists, and general food questionnaires. The table lists the countries involved, sample sizes ranging from 32 to 71,971, and various validation methods. Administration methods include interviews in person, online, and self-administered in person or online. The person reporting includes parents, children, adolescents, or both. The period of assessment varies from one day to 12 months.

Navigate back to Table 1..

## Figure 3. Long description

The map illustrates the number of dietary assessment tools (DATs) distributed across various countries in Latin America. Each country is labeled with the number of DATs it has. For example, Mexico has 5 DATs, Guatemala has 2, and Brazil has the highest count with 30. Countries like Belize, Nicaragua, Honduras, El Salvador, Panama, Paraguay, and others have 0 DATs. The map uses color shading to differentiate the number of DATs in each country, with darker shades indicating a higher number of tools.

Navigate back to Figure 3..

## Figure 4. Long description

A scatter plot illustrates the Kappa values across different age groups for food frequency questionnaires (FFQ). The x-axis represents age groups in years, ranging from 4 to 19, while the y-axis represents the Kappa value, ranging from 0.00 to 1.00. The bubble size indicates the mean energy intake in kilocalories (kcal). Color intensity represents the person reporting the food intake: gray for not specified, pink for children or adolescent, purple for parents, and light pink for both parents and children. Data points are labeled with specific kcal values and reference numbers. Notable clusters and patterns include higher Kappa values for certain age groups and reporting methods. All values are approximated.

Navigate back to Figure 4..

## Figure 5. Long description

The forest plot displays mean energy intake values from multiple studies, categorized by the type of dietary assessment tool (DAT) used and the age range of participants. Each study is represented by a point estimate with error bars indicating the confidence interval. The x-axis represents mean energy intake in kilocalories, ranging from 0 to 5000 kilocalories. The y-axis lists the studies along with the method used and the age group. Data points are color-coded based on validation status: gray for not validated, purple for validated, and light blue for unknown status. The plot includes studies using food records, 24-hour recalls, and food frequency questionnaires (FFQ), with age ranges spanning from 4 to 19 years. Notable clusters and outliers are visible, indicating variations in reported energy intake across different studies and methods. All values are approximated.

Navigate back to Figure 5..
