# Supplementary material for: Dietary assessment tools for children and adolescents in Latin America: a scoping review
Source: J Nutr Sci. 2026 Jun 17;15:e46. doi: 10.1017/jns.2026.10116 (PMC13279971; doi:10.1017/jns.2026.10116)
Supplement: Vidal et al. supplementary material [file S2048679026101165sup001.pdf]

Supplementary Material. Table 1 – Characteristics of included studies n=105

Reference	Country	Study design	Sample size	Method used	Participants (Age Range, Region, Mean Age in years)	Setting of interview	Where was applied	FCT used	Analysis of data
Bassett M. N et al., 2013	Argentina	cross sectional	241	FFQ	10 to 17, Not specified, NA	Not specified		INCAP, FCT Argentina	MS Access
Batrouni, L., 1993	Argentina	cross sectional	327	24h recall	6 to 10, Not specified, 6.9	Not specified		Food composition tables for use in Latin America	Computer program (not specified)
Carrera L. et al., 2019	Argentina	cross sectional	117	24h recall	6 to 11, Not specified, NA	Not specified	Not specified	National Health Minister	SARA (Sistema de Análisis y Registro de Alimentos 1.2.25)
Roman M. D., et al., 2021	Argentina	validation study	113	Illustrated food checklist	9 to 12, Both, 9.8	In person		not specified	
Perez-Cueto et al., 2009	Bolivia	observational	45	24h recall	12 to 16, Rural, 17.5	In person	Not specified	Bolivian FCT	
Perez-Cueto, F., Roberfroid, D. and Kolsteren, P., 2006	Bolivia	development and validation study	82	FFQ	12 to 16, Urban, NA	Not specified	School	Bolivian FCT	
Alves, M.D. et al., 2019	Brazil	cross sectional	71,298	24h recall	12 to 17, Not specified, NA	In person	Not specified	Nutrition composition table of food consumed in Brazil	ERICA-REC24h
Andrade, R.G. et al, 2003	Brazil	cross sectional	387	FFQ	12 to 17.9, Not specified, NA	Not specified		FCT in Study ENDEF	Programa de Apoio a Nutricao
Araujo M. C et al., 2010	Brazil	development and validation study	169	FFQ	12 to 19, Not specified, NA	Not specified		USDA and Brazilian FCT	Nutwin software
Araujo, M.C. et al., 2010	Brazil	development study		FFQ	12 to 19, Not specified, NA	In person	Not specified	Not specified	
Araujo, M.C., et al., 2008	Brazil	reliability study	108	FFQ	12 to 19, Not specified, 14.6	In person	Not specified	Not specified	NutWin
Assis M. A et al., 2007	Brazil	validation study	131	Illustrated food checklist	8 to 10, Not specified, 9.4	In person		Not specified	
Bagni U. V et al., 2013	Brazil	cross sectional	529	Food record	11 to 19, Not specified, NA	In person		USDA & nutrition labels	Nutwin software
Barros, M.V et al., 2007	Brazil	validation study	69	FFQ	7 to 10, Urban, NA	In person	School	Not specified	

Reference	Country	Study design	Sample size	Method used	Participants (Age Range, Region, Mean Age in years)	Setting of interview	Where was applied	FCT used	Analysis of data
Bogea E. G. et al., 2021	Brazil	cross sectional	391	FFQ	17 to 18, Urban, NA	In person		Not specified	
Bogea E. G. et al., 2021	Brazil	cross sectional	152	FFQ	17 to 18, Urban, 18.2	Online	Not specified	Not specified	REDCap
Brito Beck Da silva et al., 2015	Brazil	community trial	833	FFQ	10 to 17, Urban, NA	Not specified	Not specified	Not specified	
Carvalho, M.A, et al., 2015	Brazil	validation study	41	24h recall	7 to 10, Urban, 8.9	Online	School	Not specified	own system
Costa, M.C.D. et al., 2007	Brazil	cross sectional	2,407	FFQ	14 to 19, Not specified, 16	Not specified		Not specified	
Davies, V.F, et al., 2015	Brazil	validation study	602	Illustrated food checklist	7 to 10, Urban, 9.5	Online	School	Not specified	
Del Pino, D.L and Friedman, R., 2011	Brazil	adaptation and validation study	91	FFQ	6 to 10, Not specified, 8.17	Not specified	School	USDA	NutriBase 7 clinical softwate v.17.0
Enes, C.C and Slater, B., 2015	Brazil	cross sectional	476	FFQ	>10, Both, 11.1	Not specified	Not specified	Not specified	
Engel, R. et al., 2017	Brazil	validation study	312	Illustrated food checklist	7 to 12, Not specified, NA	Online	School	Not specified	nor specified
Freitas, J.V. et al., 2022	Brazil	development study		24h recall	4 to 9, Not specified, NA	Online	Home	Brazilian food databases and National dietary surveys (INA, ENANI and PNAE)	
Fumagalli, F., et al., 2008	Brazil	validation study	151	FFQ	5 to 10, Urban, 7.8	In person	Home	Brazilian FCT & USDA	Dietsys 4.02 software and Nutri 2.5a (Nut-DOS software)
Garcez, M.R. et al., 2021	Brazil	cross sectional	419	24h recall	12 to 19, Urban, NA	In person	Home	USDA & TACO (Brazilian food composition tables)	Nutrition Data System for Research (NDSR 2014)
Garcia, G.C. et al., 2003	Brazil	cross sectional	153	24h recall	10 to 14, Not specified, NA	In person	4	Not specified	Software Diet Win
Henn, R.L et al., 2010	Brazil	development and validation study	125	FFQ	12 to 19, Urban, 15	In person	Home	Table for Evaluation of Food Composition in	Apoio a Nutricao (Center for Health informatics)

Reference	Country	Study design	Sample size	Method used	Participants (Age Range, Region, Mean Age in years)	Setting of interview	Where was applied	FCT used	Analysis of data
								Household Measures and food labels	
Hillesheim, E., et al., 2021	Brazil	validation study	202	FFQ	9 to 13, Urban, 11.5	Not specified	Not specified	Not specified	Nutrition Data System for Research (NDSR) version 15
Jesus, G.M. et al., 2017	Brazil	validation study	390	Illustrated food checklist	6 to 15, Not specified, 9.5	Online	School	Not specified	
Jurrema-Santos, G.C., et al., 2022	Brazil	cross sectional	153	FFQ	7 to 10, Not specified, NA	Not specified	School	Brazilian Food Composition Tables (TACO: Tabela brasileira de composicao de alimentos)	ADS Nutri software (Nutrition system version 9.0, 2006, Brazil)
Kupek, E., and De Assis, M.A.A, 2016	Brazil	cross sectional	602	Illustrated food checklist	7 to 11, Not specified, 9.5	Online	School	Not specified	
Kupek, E., et al 2016	Brazil	validation study	629	Illustrated food checklist	7 to 11, Not specified, 9.5	Online	School	No use of FCT	No analysis
Leal, G.V., et al., 2010	Brazil	cross sectional	228	24h recall	10 to 18, Not specified, NA	In person	School	Table of food composition: support for nutritional decisions and nutritional labels	Virtual Nutri version 1.0 software
Leme, A.C.V and Philippi, S.T, 2017	Brazil	cross sectional	253	FFQ	14 to 18, Not specified, NA	In person	School	Brazilian Food Tables	Not specified
Lima, Scvc., et al., 2013	Brazil	cross sectional	432	24h recall	10 to 19, Not specified, NA	Not specified	School	TACO: Tabelta de composicao de alimentos 2nd Ed & USDA	Virtual Nutri Plus software
Marchioni, D.M.; et al., 2007	Brazil	reproducibility study	49	FFQ	16 to 19, Not specified, NA	Not specified	School	Brazilian FCT & USDA	Virtual Nutri version 1.0 software
Martinez, F. et al., 2013	Brazil	cross sectional	109	FFQ	15 to 19, Not specified, 16	Not specified	Not specified	Not specified	Virtual Nutri Plus software
Mascarenhas, J. M. O. et al., 2014	Brazil	development and validation study	70	FFQ	11 to 17, Not specified, 14	In person		Not specified	Virtual Nutri version 1.0 software

Reference	Country	Study design	Sample size	Method used	Participants (Age Range, Region, Mean Age in years)	Setting of interview	Where was applied	FCT used	Analysis of data
Mascarenhas, J. M. O. et al., 2017	Brazil	cross sectional	1,027	FFQ	11 to 17, Urban, 13	In person		Not specified	
Matos, S.M, et al., 2012	Brazil	validation study	108	FFQ	4 to 11, Not specified, 6.7	Not specified	Not specified	Brazilian Food composition Table (TACO), Assessment table for food consumption from home-cooking measurements and ENDEF food composition table.	Diet-pro software
Melo, A.S., et al., 2021	Brazil	cross sectional	804	24h recall	14 to 19, Urban, 16.1	In person		Table of nutritional composition of foods consumed in Brazil (Households Budget survey) and nutrition labels	Not specified
Neta, A., et al., 2021	Brazil	longitudinal epidemiological	1,431	24h recall	10 to 14, Urban, NA	In person	School	Not specified	Virtual Nutri Plus software
Oliveira, G. A., et al., 2023	Brazil	development and validation study	32	Illustrated food checklist	6 to 10, Not specified, 8	Online	Not specified	Not specified	
Oliveira, R., Peter, N., Muniz, L., 2021	Brazil	cross sectional	462	24h recall	11 to 19, Rural, 13.1	In person		Brazilian FCT (Tabela Brasileira de Composicao de Alimentos) & USDA	Nutriquanti
Pereira, L.J., et al., 2020	Brazil	longitudinal	6,349	Illustrated food checklist	7 to 12, Urban, NA	Online		Not specified	
Philippi S. and Leme A., 2015	Brazil	RCT	253	FFQ	13 to 18, Rural, 16.05	Not specified		Not specified	
Prado B. et al., 2015	Brazil	cross sectional	201	24h recall	7 to 10, Not specified, NA	In person	School	Not specified	online multiple source method
Rinaldi A. E. M. et al., 2024	Brazil	cross sectional	1,022	FFQ	10 to 19, Both, NA	In person	School	Not specified	

Reference	Country	Study design	Sample size	Method used	Participants (Age Range, Region, Mean Age in years)	Setting of interview	Where was applied	FCT used	Analysis of data
Scagliusi, F. B. et al., 2011	Brazil	cross sectional	61	FFQ	6 to 9, Urban, 7.29	In person		World Food database and Brazilian Food Composition Table	Dietsys software version 4.01
Schneider, B.C., et al., 2016	Brazil	development study	4,072	FFQ	15 to 18, Urban, NA	In person	Hospital or multiple Centre	Brazilian Table of Food Composition & USDA	Own/internal software
Silveira et al., 2023	Brazil	psychometric	2,515	FFQ	18 to 19, Urban, NA	Not specified	Not specified	Not specified	
Slater et al., 2003	Brazil	validation study	79	FFQ	14 to 18, Urban, 15.8	In person		Not specified	Virtual Nutri program
Souza R et al., 2015	Brazil	national survey	6,797	Food record	10 to 18, Not specified, NA	Not specified		compiled nutritional database (Brazilian FCT and Nutrition Coordination Center Nutrient Databank)	
Souza et al., 2016	Brazil	National Survey	71,971	24h recall	12 to 17, Urban, NA	In person	Not specified	Tabela de composicao nutritional dos alimentos consumidos no Brasil and Brazilian portion size table)	personal digital assistant (PDA) - software not specified
Sperandio, N., et al., 2016	Brazil	national survey	5,975	Food record	>10, Urban, NA	In person	Not specified	Brazilian Food composition table and USDA	computer program
Toral et al., 2007	Brazil	cross sectional	390	FFQ	>10, Urban, 12.4	In person		USDA	Dietsys Software 4.01 and EpiData 3.02
Verly et al., 2010	Brazil	cross sectional	273	24h recall	12 to 19, Urban, 17.8	In person	Home	USDA	Nutrition Data System for Research software v.2007
Voci M. et al., 2011	Brazil	cross sectional	74	FFQ	10 to 14, Urban, NA	Not specified		USDA	Dietsys software 4.01
Voci, S.M, et al., 2008	Brazil	validation study	93	FFQ	11 to 15, Urban, 13	In person		Not specified	Dietsys version 4.01 software
da Assis, M.A. et al., 2009	Brazil	validation study	164	Illustrated food checklist	6 to 11, Not specified, 8.6	In person	School	Not specified	

Reference	Country	Study design	Sample size	Method used	Participants (Age Range, Region, Mean Age in years)	Setting of interview	Where was applied	FCT used	Analysis of data
da Costa, F. and Liparotti, J., 2010	Brazil	validation study	101	FFQ	6 to 11, Urban, 9.41	In person	School	Not specified	
da Costa, F., et al., 2013	Brazil	usability study	114	Illustrated food checklist	6 to 12, Not specified, 9.2	Online	School	Not specified	not used
da Silva, E.C., et al., 2022	Brazil	cross sectional	964	FFQ	18 to 19, Not specified, NA	Not specified	Not specified	Brazilian FCT and Nutritional composition table of foods consumed in Brazil	Virtual Nutri
da Silva, J.Vl. et al., 2010	Brazil	cross sectional	272	24h recall	<16, Not specified, NA	In person	Home	Not specified	Virtual Nutri 1.0
da Veiga G. et al., 2013	Brazil	National Survey	6,797	Food record	10 to 18, Not specified, NA	In person	Not specified	Brazilian FCT & Nutrition Data System for Research database	Computer program (not specified)
de Arruda Neta A. et al., 2021	Brazil	longitudinal	1,438	24h recall	10 to 14, Not specified, 11.57	In person	School	Not specified	virtual Nutri Plus software
de Asis, M.M., et al., 2008	Brazil	reliability study	227	Illustrated food checklist	7 to 10, Not specified, 8.9	In person	School	Not specified	
de Asis, M.M., et al., 2017	Brazil	cross sectional	302	Food record	15 to 17, Not specified, 16.03	Not specified	Not specified	Tabela de composicao de alimentos: suporte para decisao nutricional	software DietPro version 4.0
de Larcerda, A.T., et al., 2023	Brazil	cross sectional	797	24h recall	8 to 12, Not specified, 9.8	In person	Not specified	Brazilian FCT	STATA
de Pinho, L., et al., 2014	Brazil	cross sectional	535	FFQ	11 to 17, Not specified, 13.43	Not specified	School	Not specified	software Diet Pro
de Vasconcelos, T.M., et al., 2016	Brazil	cross sectional	433	Food record	12 to 19, Not specified, 16.9	Not specified	Not specified	USDA & National FCT	NutWin - Programa de Apoio a Nutricao
Bel-Serrat, S., et al., 2017	Brazil & Mexico	adaptation study		24h recall	NA, Not specified, NA	Online		USDA, TACO & INSP nutritional value of foods compilation database	

Reference	Country	Study design	Sample size	Method used	Participants (Age Range, Region, Mean Age in years)	Setting of interview	Where was applied	FCT used	Analysis of data
Garcia, V. et al., 2002	Chile	cross sectional	768	24h recall	6 to 9, Rural, 7.62	In person	Hospital or multiple centre	Chilean Chemical Food Composition Table	Food processor version 6.0
Lera, L., et al., 2015	Chile	development and validation study	90	Food questionnaire	8 to 11, Not specified, NA	In person	School	Not specified	
Martinez-Arroyo, A., et al., 2022	Chile	cross sectional	576	24h recall	12 to 16, Not specified, 12.1	In person		Chilenean food composition tables, brand local food & USDA	Nutrition Data System for Research software v.2014
Herran, O., et al., 2017	Colombia	cross sectional	505	24h recall	<18, Both, 9.3	Not specified		Compilation of 4 FCT of Colombia	FoodCalc 1.3
Restrepo S. L. et al., 2023	Colombia	cross sectional	793	24h recall	14 to 20, Urban, NA	In person		Colombian specific FCT	Evaluation of Dietary Intake (EVINDI) v5 software and Personal Computer Software for Intake Distribution Estimation (PC-SIDE) v1
Rojas L. Z., et al., 2020	Colombia	cross sectional	1,282	FFQ	6 to 10, Both, 8.4	In person		Not specified	Ceres version 1.02 "FAO 1998"
Monge-Rojas, R. 2001	Costa Rica	not specified	328	Food record	12 to 19, Both, 15	Not specified		Not specified	Food processor software ESHA Research v.6
Morejon, Y., et al., 2021	Ecuador	development and validation study	100	FFQ	6 to 8, Rural, 8.58	In person		LATINFOODS (Tabla de composición de alimentos naturales y procesados para Latinoamérica)	Microsoft Excel 2013
Wang,Y., et al., 2023	Ecuador	cross sectional	276	FFQ	6 to 12, Urban, NA	Not specified		USDA	not specified
1 Macinkevage, J., et al., 2015	Guatemala	development and validation study	46	FFQ	6 to 11, Both, 8.5	In person	Home	Food composition table of INCAP	Microsoft Excel
2 Macinkevage, J., et al., 2015	Guatemala	development and validation study	49	FFQ	6 to 11, Both, 8	In person	Home	Food composition table of INCAP	
3 Macinkevage, J., et al., 2015	Guatemala	development and validation study	50	FFQ	6 to 11, Both, 8.2	In person	Home	Food composition table of INCAP	

Reference	Country	Study design	Sample size	Method used	Participants (Age Range, Region, Mean Age in years)	Setting of interview	Where was applied	FCT used	Analysis of data
Vossenaar M. et al., 2007	Guatemala	cross sectional	449	Food record	8 to 10, Urban, NA	In person		USDA and Central America FCT	
Barquera S. et al., 2003	Mexico	National Survey	2,449	24h recall	5 to 11, Not specified, NA	Not specified		INCAP, USDA, Salvador Zubirán	
Denova-Gutiérrez, E. et al., 2016	Mexico	validation study	178	FFQ	12 to 19, Both, 15.5	Online	Home	INSP food composition tables compiled	Microsoft Visual FoxPro 7.0
Esquer, M. D. P. et al., 2015	Mexico	cross sectional	552	24h recall	15 to 19, Not specified, 16.22	In person	School	INN food composition tables, handbook 8, ESHA and regional dishes	Microsoft Excel
Flores, M. et al., 2009	Mexico	national survey	8,716	FFQ	5 to 11, Both, 8.21	In person	Not specified	INSP food composition tables compiled	Not specified
Gaona-Pineda, E.b. et al., 2018	Mexico	national survey	1,961	FFQ	12 to 19, Both, 15.75	Not specified	Home	INSP food composition tables compiled	Access with SQL
Gutierrez-Pliego, L.E. et al., 2016	Mexico	cross sectional	373	FFQ	14 to 16, Not specified, 14.5			Not specified	Not specified
Oviedo-Solis, C., et al., 2022	Mexico	development and validation study	382	FFQ	5 to 19, Both, 11.36	In person	Not specified	INSP food composition tables compiled	Visual FoxPro, V7 ENSANUT 2012 software(for data entry)
Perichart-Perera, O., et al., 2010	Mexico	cross sectional	228	24h recall	9 to 13, Urban, 10.3	In person		Food processor software ESHA Research v.8 and Mexican FCT	Food processor software ESHA Research v.8
Rodriguez-Ramirez S., et al., 2009	Mexico	cross sectional	8,442	FFQ	12 to 19, Both, 15.16	Not specified		INSP food composition table	MS Access
Togo-Luna et al., 2016	Mexico	cross sectional	196	24h recall	5 to 12, Both, 8.1	In person		INCMNSZ and SMAE	Nutrickal
Zamora-Gasga V.M. et al., 2017	Mexico	cross sectional	724	Food record	9 to 12, Both, 10.7	Not specified		DIAL	DIAL dietary analysis software
Ballonga Paretas et al., 2017	Peru	cross sectional	167	FFQ	6 to 12, Rural, 8.9	In person	Home	French FCDB REGAL & Peruvian FCDB	

Reference	Country	Study design	Sample size	Method used	Participants (Age Range, Region, Mean Age in years)	Setting of interview	Where was applied	FCT used	Analysis of data
Paretas, C., et al., 2017	Peru	descriptive	171	24h recall	6 to 12, Rural, 8.9	In person	Not specified	FCT REGAL francesa & Peruvian FCT	
Rodriguez C.A, et al., 2017	Peru	validation study	118	FFQ	<15, Urban, 7.59	Not specified		Centro Nacional de Alimentación y Nutrición (CENAN) FCT & NDSR	Nutrition Data System for Research software v.2016
Rendo-Urteaga T. et al., 2019	South America (Argentina, Brazil, Colombia, Chile, Peru, Uruguay)	multicenter observational	496	FFQ	3 to 18, Both, 10.14	Not specified		USDA and local FCT	SAYCARE platform
Saravia, L., et al., 2018	South America (Argentina, Brazil, Colombia, Chile, Peru, Uruguay)	observational	702	FFQ	3 to 17, Not specified, NA	Not specified		Not specified	
Koncke, F., et al., 2022	Uruguay	cross sectional	332	24h recall	4 to 12, Not specified, NA	In person	School	Uruguayan Food composition table, Brazilian Table of Food Composition and Spanish Table of Food Composition	Diet Intake Evaluation Program (EVINDI v4)
Colina D. 2018	Venezuela	cross sectional	195	24h recall	6 to 15, Not specified, 26.39	Not specified	Not specified	FCT from Instituto Nacional de Nutricin (INN) v.1999	
Del Real, S. I., et al 2005	Venezuela	Not specified	438	24h recall	4 to 14, Not specified, 7.9	In person	Hospital or multiple Centre	Venezuelan Food composition table	
